# Spatiotemporal Drivers of the African Swine Fever Epidemic in Lao PDR

**DOI:** 10.1155/2023/5151813

**Published:** 2023-05-10

**Authors:** Ka Yi Hui, Nina Matsumoto, Jarunee Siengsanan-Lamont, James R. Young, Syseng Khounsy, Bounloum Douangneun, Watthana Thepagna, Phouvong Phommachanh, Stuart D. Blacksell, Michael P. Ward

**Affiliations:** ^1^University of Sydney, Sydney School of Veterinary Science, Camden, NSW, Sydney, Australia; ^2^Mahidol—Oxford Tropical Medicine Research Unit (MORU), Faculty of Tropical Medicine, Mahidol University, Bangkok, Thailand; ^3^National Animal Health Laboratory, Department of Livestock and Fisheries, Ministry of Agriculture and Forestry, Vientiane, Lao PDR; ^4^Centre for Tropical Medicine and Global Health, Nuffield Department of Medicine, University of Oxford, Oxford, UK

## Abstract

African swine fever (ASF) is a devastating transboundary disease of swine. Following the first report of ASF in China in August 2018, ASF spread through South-East Asian nations in 2019. Without control and containment measures, ASF can decimate smallholder pig holdings and disrupt value chains. This study aimed to describe the spatiotemporal spread of the 2019 Lao PDR ASF epidemic in domestic pigs, identify environmental (protected areas and forests), production (pig ownership), transportation (roads), and social (poverty and ethnicity) risk factors, and recommend measures that could reduce ASF spread. A retrospective spatiotemporal study was conducted at the village level. Information on the date that ASF was first reported from each case location was collected, and the outcome variable of interest “epidemic day” was created. Risk factor information from different sources was extracted for each case location. The association or correlation between epidemic day and risk factors for the spread of ASF was investigated using Kruskal–Wallis tests and Spearman rank correlation statistics. The epidemic started on June 16 and lasted for 190 days, displaying a right-skewed epidemic curve. The directional distribution was rotated approximately 305°, from Southeast to Northwest Laos. Significant risk factors for ASF associated with epidemic day were location in terms of distance from the closest protected natural area (*P* = 0.020), pig ownership (*P* = 0.005), road networks (*P* = 0.003), and poverty indices (*P* < 0.001). Cases were reported earlier in this epidemic at locations that were closer to protected natural areas, of higher pig ownership, more connected via the national road network, and which experienced elevated poverty. The spatiotemporal pattern described suggests that ASF was introduced via infected pigs from Vietnam. Based on study findings, recommendations for smallholder pig production in Southeast Asia include improving knowledge of swine value chains to inform disease risk and control, monitoring pig transportation, implementing stricter biosecurity measures on the domestic pig population, and providing biosecurity support and education to smallholder pig farmers in poverty.

## 1. Introduction

African swine fever (ASF) was first described as a disease causing high mortality events among domestic pigs in East Africa in the early twentieth century [1]. ASF virus (ASFV) first spread from the African continent to Portugal through infected pork in food waste in 1957 but was quickly eradicated [2]. However, it re-entered Portugal in 1960, and the subsequent outbreak involved several European countries [3–6]. It also spread to North and South America in the 1970s and 80s [7–9]. ASFV was eventually eliminated from these European and American countries in the late 1980s, except for the Italian island of Sardinia, where the disease became endemic for 40 years [10, 11] with control achieved from 2017 onwards with the implementation of multiple elimination programs [10, 12]. The distribution of ASFV expanded throughout Africa during the late 1990s to mid-2000s and it remains endemic [13]. With an incursion into the Republic of Georgia in 2007, ASFV quickly spread west into the European Union [14, 15] and began spreading east into Asia starting in 2014 [16–20]. The rapid spread of ASFV through Asia followed a northwest-southeast direction; it first appeared in China in 2018 and quickly spread through Southeast Asia by the end of 2019 [20].

ASF is a moderately contagious disease in swine populations [21]. It is a notifiable disease according to the World Organisation for Animal Health (WOAH), with restrictions on pork trade implemented in affected countries [22]. ASFV is a large, double stranded DNA virus that replicates mainly in the cell cytoplasm. It is the only member of the Asfarviridae family, genus *Asfivirus*. Pigs of all age groups are susceptible to ASF [21]. The clinical presentations and pathological lesions differ according to the virulence of the ASFV isolate; these are graded as high, moderate, or low virulence [22–24]. The clinical signs seen range from peracute, acute, subacute to chronic [22]. Case fatality rates in the subacute form depend on the age group of pigs. Younger pigs tend to show more severe clinical signs and will have case fatality rates of 70% to 80%; older pigs show less severe clinical signs and have case fatality rates less than 20% [25]. The case fatality rate is low in pigs with chronic ASF [25]. Signs can be nonspecific, with differential diagnoses including classical swine fever, porcine reproductive and respiratory syndrome and acute salmonellosis.

Direct contact with infected pigs, their excretions, and secretions can cause ASFV infection in susceptible animals. ASFV can infect pigs via the feeding of contaminated pig products [9]. It also spreads across country borders via wild boar or the transport of infected pigs and pig products that are subsequently consumed by susceptible pigs or wild boar [22]. *Ornithodoros* ticks can act as a source of transmission in African and European countries [26, 27]. However, the role of wild boar and ticks in ASFV transmission epidemiology within Southeast Asia requires further research [22, 28–30].

Effort has been put into developing an effective vaccine against ASFV. For instance, Vietnam's Department of Animal Health has partnered with the Agricultural Research Service of the United States Department of Agriculture to develop a recombinant vaccine [31]. However, none are approved for commercial use to date [32]. Prevention and control therefore rely on biosecurity practices, control of the movement of live pigs and trade in pig products, and culling of infected pigs. In countries such as Lao People's Democratic Republic (Lao PDR; Laos), in which the majority of pig farmers are smallholders who keep less than 5 pigs in their household [33], it is challenging to implement effective prevention and control strategies, and hence, smallholders can incur great losses.

Transmission of ASFV among swine populations can cause severe socioeconomic impacts. ASF impacts food insecurity, as pigs are an important source of protein and an efficient way to convert food waste and agricultural by-product into high quality protein. An investigation into the early Lao epidemic estimated that each affected household suffered losses of USD 215 from ASF based on the value of the pigs prior to death or slaughter, a significant financial loss for smallholders living in a region where more than 40% of the population is affected by poverty [19]. To our knowledge, research on the environmental and social drivers of ASFV spread in Laos has not been published. In a systematic review of the literature published on ASF in China, it was found that the most common cited drivers of ASFV spread were live pig transport, swill feeding, and vehicles [34]. Therefore, understanding in greater detail the role of live pig movements in the spread of ASFV can contribute to the design of better control programs. In addition, investigation of the motivators for swill feeding, such as economic circumstances, poverty and knowledge, attitude, and practices, can contribute to disease control efforts.

This study aimed to describe the spatiotemporal spread of the ASF epidemic in Laos in 2019 and to identify environmental and social risk factors (forest coverage, pig ownership, road networks, ethnicity, and poverty) for ASFV spread to inform recommendations for disease control.

## 2. Methods

### 2.1. Case Definition

A retrospective spatiotemporal study was undertaken on the spread of ASFV in Laos at the village level, based on outbreak data collected by the Department of Livestock and Fisheries (DLF) in 2019. A description of the DLF's outbreak reporting, investigation, and diagnostic confirmation process can be found in Matsumoto et al. [19]. Based on this outbreak data, the first reported outbreak was considered epidemic day 1 and each subsequent report was assigned an epidemic day relative to the primary reported outbreak. Village Chiefs and Village Veterinary Workers reported abnormal pig deaths indicating an acute or peracute ASF case to their District Agriculture and Forestry Office (DAFO), the DAFO reported to their local Provincial Agriculture and Forestry Office which then escalated the report to the national DLF to execute control measures and the National Animal Health Laboratory (NAHL) for laboratory diagnosis [19]. The NAHL used an ASFV real-time PCR to evaluate antigen presence in either whole blood, serum, or haemolysed serum [35]. A positive PCR result defined a positive case location.

### 2.2. Geocoding

Outbreak data included the names of Lao PDR provinces, districts, and villages and the latitude and the longitude of each case. Village locations were identified using information from Google Maps [36], Government of the Lao PDR's Informing Decisions for Sustainable Development's webpage [37], Lao PDR's Ministry of Agriculture and Forestry Poverty Reduction Fund webpage [38], and United States Board on Geographic Names [39]. Five cases contained village names which were not found in these sources. The village La Yao Neua could not be found directly on maps but is located within 5 km radius of another case entry at La Yao Tai. A location was randomly selected within 5 km radius of La Yao Tai. Another incomplete village entry was at Ou Nue, which could not be located in the abovementioned sources. Nevertheless, another entry was also at the village Ou Nue with a subvillage Souy Ngarm. These two entries were assumed to be related, and hence, a location was randomly generated within a 5 km radius of the Ou Nue, Souy Ngarm entry. The other three villages had names that were not found in the previous sources and were not related to other villages; hence, a village was chosen randomly within the relevant district from the gazetteer dataset produced from the United States Board on Geographic Names record [39]. One of the cases contained only the province name, so it was excluded from the spatiotemporal analysis.

### 2.3. Data Collection

An exploratory study was conducted to identify drivers of the spread of ASF in Laos. These drivers were assumed to be within the domains of the natural landscape, pig production, ethnicity, trade, and economics. Selection of these domains was based on knowledge of the spread of ASF and other transboundary diseases [21, 40] and included the susceptible species (pig production for domestic pigs and landscape for wild boar), biosecurity (poverty), and contact structures (roads and ethno-linguistic family). Environmental and social data used for risk factor analyses were accessed from official government census webpages and publicly accessible sources. The data on forest coverage and the type of forest at a district level (updated 2015) are available on the Open Development Mekong's webpage [41]. The percentage of agricultural households with pigs, the number of pigs, the incidence of poverty, the number of poor people, and the ethno-linguistic family at a village level are accessible via the Government of the Lao PDR's Informing Decisions for Sustainable Development's webpage (2011) [37]. The number of roads and the roads length data (2009) were downloaded from DIVA-GIS [42]. The risk factors investigated were chosen to broadly represent wild boar, pig production, trade, and socioeconomic factors as drivers of the spread of ASF in Laos in 2019. The definitions and sources of these data can be found in Table 1.

Data describing a total of fifteen potential environmental, economic, and social risk factors were identified from three environmental and five social related datasets. The three environmental datasets were as follows: (1) the protected areas in Laos, (2) the percentage of pig ownership at the village level, and (3) the number of pigs owned at the village level. The five social related datasets were as follows: (1) the number of roads, (2) the road length, (3) the incidence of poverty, (4) the number of poor people, and (5) the ethno-linguistic family. Data for risk factor analysis were generated via data extraction from these datasets within a GIS.

Lao PDF forests are categorised into three types; hence, the protected area risk factors consisted of three categories: (1) national protected area, (2) national protection forest, and (3) national production forest. National protected areas (previously called National Biodiversity Conservation Areas [43]) cover 3.8 million hectares [44]. According to the Forestry Law (1996) in Laos, Article 18 “Conservation forest is forest and forest land set aside for the purposes of conservation of fauna, flora, nature, and various things of historical, cultural, touristic, and environmental value and for scientific study and research.” [43] National protection forests cover 7.4 million hectares of land [44]. The Forestry Law (1996) also defines protection forests having roles as “watershed protection, erosion control, national security, and prevention of natural disasters… generally unmanaged and are the poorly defined areas in steep terrain along international borders.” [43] National production forest covers 3.1 million hectares [44] and includes natural forests and planted forests for logging and nontimber forest products as commodities to support people's livelihoods and contribute to the country's socioeconomic development [44].

Five risk factors were created from the Lao Forests dataset: (1) whether the case is located within a protected area (yes, no), (2) if the case is not located within a protected area, the distance (km) from the nearest protected area, (3) the number of protected areas within 10 km of the case location, (4) the land area (sq. km) of protected area within a 10 km radius of the case, and (5) the proportion (%) of the protected area within a 10 km radius of the case.

The proportion (%) of households with pigs was projected onto the Lao PDR shapefile. Two risk factors were extracted from this dataset: (1) pig ownership (%) in the village at the case location, and (2) average pig ownership (%) within a 10 km radius of the case location. A dataset of the number of owned pigs at the village level was used to create the sum of the number of owned pigs within a 10 km radius of the case location as a potential risk factor.

The road networks data were projected onto the Lao PDR shapefile. From these data, two risk factors were created: (1) a count of roads within a 10 km radius of each case location and (2) the total length of roads (km) within a 10 km radius of each case location.

Two risk factors were extracted from the incidence of poverty dataset: (1) the incidence of poverty at the case location and (2) the average incidence of poverty within a 10 km radius of each case location. Another dataset related to poverty was the number of poor people, and two risk factors were extracted from these data: (1) the number of poor people at each case location and (2) the average of the number of poor people within a 10 km radius of each case location.

Population by ethno-linguistic family is a categorical variable. The dominant ethno-linguistic group (more than 80% of the population) at the case location was explored as a potential risk factor.

A description of the 15 risk factors that were created for analysis is shown in Table 1. These were separated into categorical and continuous risk factor variables for analysis.

### 2.4. Spatiotemporal and Risk Factor Analysis

Data were managed in Excel (Microsoft Corporation. Microsoft Excel. 2018), which was also used to conduct simple descriptive analyses. Utilising ArcGIS Pro (ESRI Inc. ArcGIS Pro version 2.9.3. 2021), the edited data were imported and mapped onto a Lao PDR shapefile (World Geographic System 1984) [42]. Dots were used to visualise case locations, and colours were used to show the temporal distribution of locations plotted by epidemic day. Datasets describing risk factors were projected (Universal Transverse Mecator 48N) onto the Lao PDR shapefile, and then relevant information was extracted from the case locations, or within a 10 km radius of the case location using buffers, for analysis (Table 1).

### 2.5. Statistical Analysis

Data were analysed using IBM SPSS Statistics for Windows, version 28.0, 2022. In this study, epidemic day (a continuous variable, ranging from 1 to 190) was the outcome variable of interest. Values of the predictor variables were assumed to be fixed throughout the 190 days of the epidemic. Association between epidemic days and the predictor variables, including the environmental, economic, social, and production risk factors, was investigated. Descriptive analysis and independent-samples Kruskal–Wallis hypothesis tests were used to analyse the categorical data. Spearman correlation analysis was used to analyse the continuous data. The null hypothesis for this study was that ASF outbreaks occurred randomly throughout the country, and any significant association between epidemic day (outcome) and a risk factor (predictor) suggested that the risk factor might contribute to when ASF was reported and be associated with the spread of ASF throughout Laos during 2019.

## 3. Results

**Figure 1:**
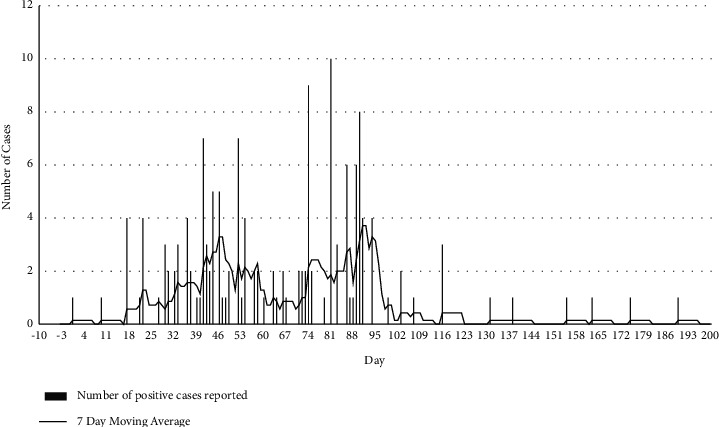
Number of African swine fever cases reported daily from 16th June (day 1) to 31st December 2019 (day 190), with a 7 day moving average showing the underlying trend of the cases.

The reported ASF epidemic lasted for 190 days (27.1 weeks), 16 June to 23 December 2019, during which period a total of 153 village cases occurred. The median epidemic day was day 65 (20 August; week 10), with an interquartile range of 42 to 86 (28 July to 10 September). The epidemic curve showed a bimodal pattern, with modes on day 45 (31 July) and day 90 (14 September). The epidemic was right-skewed (median day 65 versus 190 days epidemic length). The epidemic curve is shown in Figure 1.

**Figure 2:**
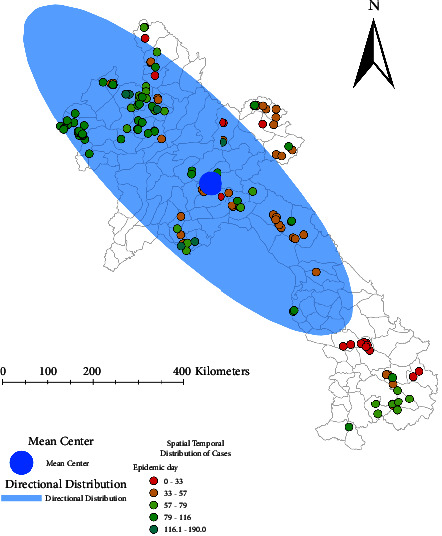
Spatial and temporal distribution of African swine fever (ASF) cases in 2019. Cases are shaded from red to green by epidemic day (1 to 190). Mean centre is represented by the blue circle and the directional distribution is represented by the light blue shade.

From the mapped cases in Laos (Figure 2), most of the first 20% of cases occurred in the Southeast part of the country, the second 20% of cases were located mainly in the centre of the country, and the final 60% of cases were mostly in the Northeastern part of Laos (with a few cases in the South-eastern part). The mean distribution of cases was located close to Xiangkhouang. The directional distribution was rotated approximately 305°, from Southeast to Northwest Laos.

Categorical variables included location within a protected area (yes, no) and ethno-linguistic family (Hmong-Mi, Lao-Tai, Mon-Khmer, Sino-Tib, and mixed). The association between these risk factors and epidemic day is shown in Table 2. Neither of the factors were significantly associated with epidemic day.

For continuous variables (Table 3), seven of these showed a significant negative correlation with epidemic day: the distance from the case location and the nearest protected area (*P*=0.020), pig ownership % at the case location (*P*=0.002), the average pig ownership % within 10 km of the case location (*P*=0.005), the number of roads within 10 km of the case location (*P*=0.003), the total length of roads within 10 km of the case location (*P*=0.003), the proportion (%) of the population living below the poverty line at the case location (*P*=0.042), and the average proportion (%) of the population living below the poverty line within 10 km of the case location (*P* < 0.001). ASF outbreaks were reported earlier in this outbreak (negative correlation coefficient) at locations closer to protected natural areas, in areas of higher pig ownership, that were more connected via the national road network and which experienced elevated poverty.

## 4. Discussion

The risk factors selected in this study were proxies for potential drivers of ASFV spread. Protected areas could be related to the presence of wild boar and free ranging pig production (in which domestic pigs are free to roam for at least part of the day), with likely lower biosecurity and thus pathways for the spread of ASFV [22, 26, 27]. Pig ownership and the number of pigs reflect domestic pig density in local areas. The number of roads and the length of roads reflect the likely connectivity, trade, and logistics present within local areas. Poverty is indicative of economic status, and ethnic groups could be related to relevant cultural factors, such as pig production systems, family and community networks, and the value chain, that potentially promote the spread of ASFV.

The number of pigs and pig ownership were significantly correlated with the epidemic day: case locations that occurred earlier in the epidemic were more likely to be in areas with more pigs and higher household pig ownership. This finding was expected, and besides having a biological interpretation (pigs being the host species for ASFV), it could indicate that cases reported during this epidemic were more focused in areas of higher pig production. A better understanding of the smallholder pig production system and value chain would contribute to insights into the spread of contagious pig diseases, such as ASF. It is also needed for effective disease surveillance.

Road data (number and length) were significantly correlated with the epidemic day: outbreaks earlier in the epidemic were more likely to occur in districts with more roads and a larger road network. This suggests that there was a possible contribution of trade and movement of pigs and pig products via the road network in the spread of ASFV during the 2019 epidemic in Lao PDR. Studies conducted at a country scale in Africa and China demonstrate the importance of road networks contributing to pandemics and should be considered when planning for prevention and control in future outbreaks [45, 46]. The role of the road network in ASFV spread requires further investigation so that it can be integrated into control and surveillance programs.

Poverty was also significantly correlated with the epidemic day: outbreaks earlier in the epidemic were more likely to occur in districts with greater poverty. Poverty is indicative of economic status and might indicate that the pig production system in these areas is more susceptible to ASFV infection due to a range of poverty-linked factors, such as poorer biosecurity, lack of education, inadequate housing, and poorer nutrition, as has been noted in other research [47, 48].

Based on the risk factors identified in this study, suggestions to control ASFV spread in Lao PDR and small holder pig production systems in other Southeast Asian countries include implementing biosecurity measures within the domestic pig population. Transportation of pigs should be monitored to avoid the spread of ASFV when pigs are infectious. If any case is detected, livestock standstill should be executed strictly since it is known to be helpful in limiting the spread of transboundary animal diseases [49]. Since we found that areas of poverty were more likely to report ASF earlier in the epidemic, providing financial, educational, and biosecurity support to households that are in poverty might help lower the risks of disease spread. Very little research on ASF in Southeast Asia, and Laos PDR in particular, has been undertaken. One reason is that the incursion of ASFV is so recent, 2019, and following that (2020–2022), research opportunities were likely limited due to the impact of the COVID pandemic. We have undertaken a hypothesis-generating study as an initial approach to understand what might have driven the spread phase of ASFV in Laos. This is a retrospective study, so information on covariates was not recorded at the time of the incursion. Rather, we relied on the measurement of covariate information from publicly available data, and we focused on covariates which do not change rapidly over time (at least in the context of the incursion and rapid spread of a pathogen such as ASFV). Follow-up research is needed to verify the role of the factors identified in this study.

Based on the reported spatiotemporal distribution of case locations, the ASF epidemic started in the Southeast of Laos and spread Northwest relatively uniformly across the country from June to December 2019. Despite this overall spread pattern, some outbreaks occurred later in areas that apparently had already become infected with ASFV. In February, the same year, Vietnam detected the first case of ASF [50], four months before the first Laos ASF report. The Lao epidemic could have potentially been due to importation of infected pigs from Vietnam because the first few cases were within the vicinity of the main road that connects Vietnam, Laos, and Thailand in the southern part of the country [36]. Thailand reported its first case of ASF in January, 2022, 2.5 years after Laos. Although there is some evidence that ASFV entered Laos from Vietnam, further investigations would be needed to strengthen this hypothesis.

One issue identified in the epidemic dataset was that the reported date of the case occurrence might not be equivalent to the actual date of first infection and disease onset. However, we expect the difference to be small and the error to be nonsystematic so that the bias is unlikely to impact the findings from this study. In this study, surveillance and reporting challenges forced animal health staff to report some cases in groups. Future investigations could incorporate the variation in report date to make analyses more accurate [19].

Another limitation is the availability of village scale maps and data on risk factors. With the frequent resettlement of rural populations in Laos [51], the rural villages shown on maps are not constant and some village names could not be found. By using the available information to identify the most precise area, random generation of locations was used to generate five cases' locations. This approach was considered to introduce minimal bias, and these villages represent only about 3% of the outbreak dataset. Risk factor data are not available every year, for example, the census data are available only every five years, so that some of the data (especially census data) might not accurately reflect the situation at the time of the epidemic. The most recent datasets were used, and an assumption was made that no large changes occurred over relatively short time period (190 days) of the epidemic. At the broad temporal scale, factors such as main roads, poverty indices, forests, and ethno-linguistic family are unlikely to change quickly. Analyses such as those conducted in this study are limited by the covariate information that is publicly available, including spatial and temporal resolution and timeliness. Therefore, the results of such analyses should be used to generate hypotheses that are further investigated in more detailed and fine scale studies, where possible. The nature of incursions of transboundary diseases such as ASF makes follow-up studies challenging to conduct. Finally, ecological bias is a risk in studies of landscape factors and disease spread. In this study, we restricted our inference to the outbreak (village) level to avoid ecological bias.

Findings from this study suggest that landscape, trade, and socioeconomic factors contribute to the spread of ASFV in smallholder systems and developing countries, such as Laos in 2019. Targeted surveillance of road networks and locations in areas of protected and natural forests, and improved biosecurity in areas of pig production which suffer from poverty, are strategies to consider. Finer scale studies on these factors are warranted because control of ASFV spread depends on addressing these drivers and developing appropriate control, prevention, and surveillance programs.

## Figures and Tables

**Table 1:** Description of 15 risk factors that were used to explore the drivers for spread of African swine fever virus in Lao PDR in 2019.

Risk factors	Variables	Levels
Protected area	Whether or not case is located within a protected area	National protected area, national protection forest, and national production forest
	If the case is not located within a protected area, distance (decimal degrees) to the nearest protected area	0 to 0.425
	The number of protected areas within 10 km of the case location	0, 1, 2, 3, 4
	Land area (km^2^) of the protected area within 10 km of the case location	0 to 339
	Proportion (%) of the protected area within 10 km of the case location	0 to 100

Households with pigs	Share of pig ownership (%) in the village at the case location	0 to 100
	The average share of pig ownership (%) within 10 km of the case location	0 to 100

Number of pigs	The sum of number of owned pigs within 10 km of the case location	0 to 9405

Roads	The number of roads within the 10 km of the case location	0 to 12
	The length (km) of roads within 10 km of the case location	0 to 81

Incidence of poverty	% Population living below the poverty line at the case location	1.32 to 82.18
	Average % population living below the poverty line within 10 km of the case location	6.61 to 73.32

Number of poor people	The number of people below the poverty line at the case location	6.59 to 1957.34
	The average number of people below the poverty line within 10 km of the case location	34.32 to 603.53

Ethno-linguistic family	Dominant (>80%) ethno-linguistic family dominated at the case location	Lao-Tai, Sino-Tibetan, Hmong-Mien, Mon-Khmer, and mixed

**Table 2:** Association between the date of reporting (epidemic day) of African swine fever (ASF) cases in the 2019 Lao PDR outbreak and location within a protected (natural) area and ethno-linguistic family (Kruskal–Wallis test).

Risk factors	Category	Sample size	Median epidemic day	Test statistic	*P*
Protected area	No	85	63	0.010	0.920
	Yes	57	66		

Ethno-linguistic family	Hmong-Mi	12	74	7.175	0.127
	Lao-Tai	44	60		
	Mixed	48	73.5		
	Mon-Khmer	41	54		
	Sino-Tib	7	81		

**Table 3:** Association between the date of reporting (epidemic day) of African swine fever (ASF) cases in the 2019 Laos outbreak and protected (natural) area, pig production, and poverty (Spearman rank correlation, *r*).

Risk factors	Min	Max	Median	IQR	Epidemic day	
					*r*	*P*
Distance to the nearest protected area	0	0.43	0.02	0.04	0.057	0.486
The number of protected areas within 10 km of the case location	0	4	2.00	1	−0.253	**0.020**
Land area (km^2^) of the protected area within 10 km of the case location	0	315	136.65	181	−0.034	0.677
The proportion of the protected area within 10 km of the case location	0	100	43.53	58	−0.041	0.616
Share of pig ownership (%) in the village at the case location	0	100	57.89	47	−0.253	**0.002**
The average share of pig ownership (%) within 10 km of the case location	1	93	57.59	26	−0.225	**0.005**
The number of pigs	0	9405	2359	2134	−0.008	0.925
The number of roads	0	12	3	3	−0.237	**0.003**
Length of roads	0	84	28.76	21	−0.242	**0.003**
% Population living below the poverty line at the case location	1	82	24.38	22	−0.166	**0.042**
The average % population living below the poverty line within 10 km	7	73	23.99	13	−0.272	**<0.001**
The number of people below the poverty line	7	1957	153.10	226	−0.125	0.125
The average number of people below the poverty line	34	604	156.21	92	−0.064	0.435

## Data Availability

Data are not freely available due to ownership by the Lao PDR Department of Livestock and Fisheries.
